# Socioeconomic Status Is Significantly Associated with the Dietary Intakes of Folate and Depression Scales in Japanese Workers (J-HOPE Study)

**DOI:** 10.3390/nu5020565

**Published:** 2013-02-18

**Authors:** Koichi Miyaki, Yixuan Song, Setsuko Taneichi, Akizumi Tsutsumi, Hideki Hashimoto, Norito Kawakami, Masaya Takahashi, Akihito Shimazu, Akiomi Inoue, Sumiko Kurioka, Takuro Shimbo

**Affiliations:** 1 Division of Clinical Epidemiology, Department of Clinical Research and Informatics, National Center for Global Health and Medicine, Toyama 1-21-1, Shinjuku-ku, Tokyo, Japan; E-Mails: isyuson@ri.ncgm.go.jp (Y.S.); taneichi.setsuko@mail.u-tokyo.ac.jp (S.T.); tshimbo@hosp.ncgm.go.jp (T.S.); 2 Office for Mental Health Support, Division for Counseling and Support, the University of Tokyo, 7-3-1 Hongo, Bunkyo-ku, Tokyo, Japan; 3 Department of Public Health, Kitasato University School of Medicine, 1-15-1 Kitasato, Minami-ku, Sagamihara, Japan; E-Mail: akizumi@kitasato-u.ac.jp; 4 Department of Health Economics and Epidemiology Research, School of Public Health, University of Tokyo, 7-3-1 Hongo, Bunkyo-ku, Tokyo, Japan; E-Mail: hidehashimoto-circ@umin.ac.jp; 5 Department of Mental Health, Tokyo University Graduate School of Medicine, 7-3-1 Hongo, Bunkyo-ku, Tokyo, Japan; E-Mails: norito@m.u-tokyo.ac.jp (N.K.); ashimazu@m.u-tokyo.ac.jp (A.S.); 6 National Institute of Occupational Safety and Health, Nagao 6-21-1, Tama-Ku, Kawasaki, Japan; E-Mail: takaham@h.jniosh.go.jp; 7 Department of Mental Health, Institute of Industrial Ecological Sciences, University of Occupational and Environmental Health, 1-1, Iseigaoka, Yahata-nishi-ku, Kitakyushu, Japan; E-Mail: akiomi-tky@umin.ac.jp; 8 Department of Health Policy and Management, University of Occupational and Environmental Health, 1-1, Iseigaoka, Yahata-nishi-ku, Kitakyushu, Japan; E-Mail: kurioka@med.uoeh-u.ac.jp

**Keywords:** socioeconomic status, education, household income, folate intake, depression

## Abstract

The association of socioeconomic status (SES) with nutrient intake attracts public attention worldwide. In the current study, we examined the associations of SES with dietary intake of folate and health outcomes in general Japanese workers. This Japanese occupational cohort consisted off 2266 workers. SES was assessed by a self-administered questionnaire. Intakes of all nutrients were assessed with a validated, brief and self-administered diet history questionnaire (BDHQ). The degree of depressive symptoms was measured by the validated Japanese version of the K6 scale. Multiple linear regression and stratified analysis were used to evaluate the associations of intake with the confounding factors. Path analysis was conducted to describe the impacts of intake on health outcomes. Education levels and household incomes were significantly associated with intake of folate and depression scales (*p* < 0.05). After adjusting for age, sex and total energy intake, years of education significantly affect the folate intake (β = 0.117, *p* < 0.001). The structural equation model (SEM) shows that the indirect effect of folate intake is statistically significant and strong (*p* < 0.05, 56% of direct effect) in the pathway of education level to depression scale. Our study shows both education and income are significantly associated with depression scales in Japanese workers, and the effort to increase the folate intake may alleviate the harms of social disparities on mental health.

## 1. Introduction

The association of socioeconomic status (SES) with health outcomes attracts public attention worldwide [1]. Health outcomes such as abdominal obesity [2], metabolic syndrome [3], hypertension [4,5], atherosclerosis [6], cardiovascular disease (CVD) [7] and depression [8,9], are more common in persons of low SES groups. Unhealthy behaviors in low SES groups are seen to be one of the mechanisms linking to a worse state of health [9,10]. Recently, many studies have shown significant differences in dietary intake and quality between levels of SES [11,12,13,14,15,16,17,18,19]. Low SES groups had a lower intake of most nutrients good for health and the gap between their intake and the dietary reference intake was wider than that for the high SES groups.

Several studies of dietary intake showed that folate intake relates to CVD [20], colorectal cancers [21], depression [22,23,24,25], cognitive function [26,27,28] in general populations. Folate intake was thought to be influenced by incomes in the general population. In a Brazilian study, newborns from mothers of low SES groups presented low folate levels than those born from high SES groups [29]. Another study from Mexico reported that intakes of most nutrients including folate were lower in low SES groups [30]. In studies of USA, the excess prevalence of low folate levels associated with the low SES groups [31,32]. Almost these previous studies focused on income or economic state [29,30,31,32], but lack of the associations with education. Although there are several studies that examine the association between education and dietary intake [15,16,17], few studies have been carried out to investigate the association of education with folate intake.

The aim of this study is to investigate the associations between SES factors, nutritional intake and health incomes in our occupational cohort, especially to identify the role of nutritional intake in these potential associations. Folate was selected as study object because it is widely used, essential to life and considered relative to many diseases. To our knowledge, this is the first study to evaluate the relations of folate intakes to income and education, as well as depression scales, in Japanese general workers.

## 2. Materials and Methods

### 2.1. Subjects

The present cross-sectional study is a part of the Japanese study of Health, Occupation and Psychosocial factors related Equity (J-HOPE), which was performed to develop and expand research to elucidate mechanisms underlying the social disparity in health and establishment of measures to control over it. It was based on a baseline survey of our occupational cohort study on social class and health, supported by a grant from the Ministry of Education, Culture, Sports, Science and Technology, Japan. A total of 14,534 individuals from 13 independent cohorts enrolled in this study. In the current investigation, employees of a Japanese major manufacturing company (Headquarter is in Kyoto and the other major 21 offices were spread all over the Japan) were recruited. Approximately 2500 workers of this company were invited to participate, and 2266 agreed (response rate 90.1%). The protocol and explanation documents of our study were approved by the ethics committee of the University of Tokyo School of Medicine, and written informed consent was obtained from each subject.

### 2.2. Measurements

Age, sex, height, weight, systolic and diastolic blood pressures (SBP and DBP), fasting plasma glucose level (FPG) and serum lipid levels (total cholesterol, triglyceride, high density lipoprotein (HDL) cholesterol) were measured at health check-ups in all subjects. Body mass index (BMI) was calculated as dividing the weight (in kg) by the square of the height (in meters). The degree of depressive symptoms was measured by the Japanese version of the Kessler’s K6 questionnaire [33], which consists of six items asking how frequently respondents have experienced symptoms of psychological distress during the past 30 days. The response options range from 0 = none of the time to 4 = all of the time, resulting in score ranges of 0–24. A cut-offs of ≥9 for the K6 for identifying subjects at high risk of depressive symptom was suggested. The Japanese version of K6 is considered to have similar screening performance and better acceptability as compared with that of the center for epidemiologic studies depression scale (CES-D) [34].

### 2.3. Socioeconomic Status (SES)

Years of education, annual household income added by income of each family member, and the numbers of family members were assessed by a self-administered questionnaire. Each participant was asked to answer which of the six income grades his/her household income belongs to, defined as: 1, <3.0 million yen/year; 2, 3.0–4.99 million yen/year; 3, 5.0–7.99 million yen/year; 4, 8.0–9.99 million yen/year; 5, 10.0–15.0 million yen/year; 6, >15.0 million yen/year. The midpoint of each grade was calculated (1, 1.5 million yen/year; 2, 4.0 million yen/year; 3, 6.5 million yen/year; 4, 9.0 million yen/year; 5, 12.5 million yen/year; 6, 20.0 million yen/year). The classification of education subgroups is based on the International Standard Classification of Education (ISCED) (approved by the United Nations Educational Scientific and Cultural Organization (UNESCO) General Conference at its 29th session in November 1997): “Low” education level corresponds to the end of compulsory education, and can also include vocational training after schooling (less than 12 years, ISCED level 1 and 2); “Middle” education level corresponds to at least three years of additional schooling, which includes programs designed to provide access to higher education or leads directly to the labor market (12–15 years, ISCED level 3 and 4); “High” education level corresponds to a Bachelor’s degree or higher (≥16 years, ISCED level 5 and 6).

### 2.4. Dietary Intake

Dietary habits during the preceding month were assessed with a validated, brief, self-administered diet history questionnaire (BDHQ) [35]. 

Responses to the BDHQ were checked for completeness and, where necessary, clarified by direct questioning of the subject. The BDHQ is a four-page structured questionnaire that enquires about the consumption frequency of a total of 56 food and beverage items, with specified serving sizes described in terms of the natural portion or the standard weight and volume measurement of servings commonly consumed in general Japanese populations. The BDHQ was developed based on a comprehensive (16-page) version of a validated self-administered diet history questionnaire [36,37,38]. The BDHQ includes the main food sources for Japanese with regard to folate (vegetables [nine items: lettuce; tomatoes; dark-green leafy vegetables; cabbage; carrots and pumpkins; radishes and turnips; onions, burdock, and lotus roots; mushrooms; and seaweed] and green tea (one item)). The validation of the BDHQ was performed by using 16-day weighed dietary records as the gold standard, Pearson correlation coefficients for folate intake in 92 Japanese men and 92 Japanese women aged from 31 to 76 years were 0.50 and 0.62, respectively [35]. Adjusted folate intakes were calculated as daily folate intakes divided by daily total energy intakes (1000 kJ).

### 2.5. Statistical Analysis

We aimed to evaluate the relationships between folate intakes and SES factors. Descriptive statistics of clinical characteristics among different education or income groups were compared. Pearson’s correlation coefficients and *p* values represent the relationships between SES factors and intake levels. The association between intake levels of folate and SES factors was examined by multiple linear regression analyses, controlling for age, sex and total energy intakes. The total subjects were stratified into SES subgroups, we calculated age-, sex- and total energy intake-adjusted intake level for folate of each subject, and compared the mean adjusted values between subgroups by using Bonferroni-corrected trend test. Finally, the structural equation modeling (SEM) analysis was performed to estimate the causal relationship between the SES factors and depressive score. The IBM SPSS statistics for Windows version 19.0J (IBM, Armonk, NY, USA) and AMOS 19.0 (IBM, Armonk, NY, USA) statistics software packages were used for all statistical analyses. Statistical significance for all analyses was defined as *p* < 0.05. 

## 3. Results

Table 1, Table 2 illustrate the basic characteristics, SES factors and intake levels of the participants stratified by education (Table 1) or income level (Table 2). The mean (±standard deviation, SD) age and BMI of the total subjects (*n* = 2266) were 43.4 ± 9.8 years (ranged from 21 to 65 years) and 23.1 ± 3.3 kg/m^2^ (ranged from 13.8 to 41.8 kg/m^2^), respectively, which are typical for middle-aged Japanese population. Two hundreds and forty-one of them are women, accounting for 10.6%. 63.6% of the subjects (*n* = 1442) attained the Japanese RDA of folic acid intake (240 μg/day), and 824 did not.

**Table 1:** Clinical characteristics, dietary nutrients intake data, and socioeconomical status (SES) factors of the study subjects according to different education level groups.

	Total subjects (*n* = 2266)	Low education level group (*n* = 131)	Middle education level group (*n* = 943)	High education level group (*n* = 1192)	*p* for trend adjusted for age and sex
Age (year)	43.4 ± 9.8	51.6 ± 9.7	45.6 ± 9.0	40.9 ± 9.4	<0.001 **
Proportion of women (%)	10.6	5.3	16.3	6.7	<0.001 **
*Clinical characteristics*					
Body mass index (kg/m^2^)	23.1 ± 3.3	23.3 ± 3.7	23.0 ± 3.4	23.1 ± 3.1	0.287
Serum total cholesterol (mg/dL)	200.0 ± 35.1	209.0 ± 34.0	199.2 ± 33.5	199.6 ± 36.6	0.831
Serum triglyceride (mg/dL)	125.8 ± 180.0	142.4 ± 109.9	120.5 ± 89.7	128.8 ± 242.4	0.977
Serum HDL cholesterol (mg/dL)	61.8 ± 16.5	59.8 ± 17.0	62.9 ± 17.1	61.0 ± 15.8	0.679
Fasting plasma glucose (mg/dL)	95.0 ± 23.2	100.7 ± 25.8	95.4 ± 25.3	94.2 ± 20.6	0.245
Depression scale (K6 score)	5.1 ± 4.6	4.9 ± 5.0	5.3 ± 4.7	5.1 ± 4.6	0.032 *
*SES factors*					
Years of education (year)	14.5 ± 2.5	9.4 ± 0.7	12.6 ± 0.9	16.7 ± 1.0	<0.001 **
Proportion of manager (%)	22.7	10.7	9.4	34.6	<0.001 **
Annual household income (ten thousands yen/year)	704.4 ± 297.5	656.9 ± 336.4	665.9 ± 255.9	740.1 ± 318.6	<0.001 **
*Dietary intake levels*					
Total energy intake (kJ/day)	7705.8 ± 2396.0	7527.6 ± 2560.4	7423.5 ± 2333.0	7948.6 ± 2402.3	<0.001 **
Total energy adjusted folate intake (μg/1000 kJ·day)	39.0 ± 13.6	37.7 ± 14.0	38.2 ± 14.7	39.7 ± 12.5	<0.001 **

Values are shown as mean ± standard deviation or percentage; The classification of education subgroups is based on the International Standard Classification of Education (ISCED) 1997; Subjects with low (<12 years), middle (12–15 years) and high (≥16 years) levels of education were compared; For continuous variables we used linear regression analysis adjusted for age and sex; For categorized variables, we used logistic regression analysis adjusted for age and sex; * *p* < 0.05, ** *p* < 0.01.

**Table 2:** Clinical characteristics, dietary nutrients intake data, and socioeconomical status factors of the study subjects according to different household income groups.

	Total subjects (*n* = 2266)	Group 1 (*n* = 76)	Group 2 (*n* = 472)	Group 3 (*n* = 1049)	Group 4 (*n* = 403)	Group 5 (*n* = 239)	Group 6 (*n* = 26)	*p* for trend adjusted for age and sex
Age (year)	43.4 ± 9.8	34.5 ± 13.5	36.6 ± 11.0	44.0 ± 7.7	48.6 ± 7.3	48.4 ± 7.7	48.9 ± 7.7	<0.001 **
Proportion of women (%)	10.6	31.6	13.8	7.0	7.7	18.0	15.4	<0.001 **
*Clinical characteristics*								
Body mass index (kg/m^2^)	23.1 ± 3.3	21.8 ± 3.5	22.7 ± 3.4	23.3 ± 3.3	23.1 ± 3.0	23.3 ± 2.9	24.6 ± 3.6	0.049 *
Serum total cholesterol (mg/dL)	200.0 ± 35.1	186.4 ± 26.1	193.3 ± 36.0	200.9 ± 36.5	202.4 ± 31.5	201.9 ± 33.0	199.6 ± 34.5	0.132
Serum triglyceride (mg/dL)	125.8 ± 180.0	100.0 ± 51.3	116.3 ± 85.6	130.2 ± 230.1	123.5 ± 94.1	120.5 ± 130.5	166.0 ± 149.3	0.866
Serum HDL cholesterol (mg/dL)	61.8 ± 16.5	60.4 ± 13.3	62.3 ± 16.3	61.6 ± 16.7	61.3 ± 16.3	63.6 ± 16.7	58.7 ± 16.4	0.931
Fasting plasma glucose (mg/dL)	95.0 ± 23.2	89.5 ± 23.4	92.2 ± 21.8	95.2 ± 23.6	97.1 ± 22.2	93.7 ± 20.2	103.2 ± 45.8	0.954
Depression scale (K6 score)	5.1 ± 4.6	5.3 ± 5.1	5.6 ± 4.9	5.3 ± 4.7	4.9 ± 4.5	4.3 ± 3.9	3.7 ± 3.9	0.023 *
*SES factors*								
Years of education (year)	14.5 ± 2.5	14.1 ± 2.9	14.6 ± 2.7	14.3 ± 2.6	14.8 ± 2.3	15.2 ± 2.0	15.6 ± 3.5	<0.001 **
Proportion of manager (%)	22.7	0	2.5	13.1	50.9	60.3	65.4	<0.001 **
Annual household income (ten thousands yen/year)	443.0 ± 188.5	127.5 ± 29.3	312.2 ± 87.1	415.6 ± 123.2	522.5 ± 129.3	720.5 ± 151.5	1078.3 ± 167.6	<0.001 **
*Dietary intake levels*								
Total energy intake (kJ/day)	7705.8 ± 2396.0	7424.5 ± 2763.3	7496.8 ± 2461.2	7780.5 ± 2427.8	7827.8 ± 2209.2	7717.7 ± 2310.5	7389.8 ± 2196.8	0.022 *
Total energy-adjusted folate intake (μg/1000 kJ·day)	39.0 ± 13.6	38.2 ± 14.7	37.5 ± 13.9	38.6 ± 13.9	39.4 ± 12.3	41.9 ± 12.7	46.4 ± 14.6	0.008 **

Values are shown as mean ± standard deviation or percentage; Subjects are classified into 6 grades according to their self-reported household incomes (1, <3.0 million yen/year; 2, 3.0–4.99 million yen/year; 3, 5.0–7.99 million yen/year; 4, 8.0–-9.99 million yen/year; 5, 10.0–15.0 million yen/year; 6, >15.0 million yen/year) and are compared among different grades; The income data for one person is missing; For continuous variables we used linear regression analysis adjusted for age and sex; For categorized variables, we used logistic regression analysis adjusted for age and sex; * *p* < 0.05, ** *p* < 0.01.

The correlations of two major SES factors, education and household income, with dietary intakes of folate, were evaluated and the results are present in Table 3. Folate intake levels positively related to both years of education and household income as continuous variable, Pearson’s correlation coefficients (*R*) were 0.074 (*p* value <0.001) and 0.101 (*p* value <0.001), respectively. When the univariate analysis was used, the associations of total energy-adjusted folate intake with SES were also present, and the unadjusted *p* value for years of education was 0.029, for household income was below 0.001.

**Table 3:** The associations of daily dietary intakes of folate with SES factors (years of education and adjusted annual household income).

	Relations of SES factors with total energy adjusted folate intakes		Associations of folate intakes with related factors	
	Pearson’s correlation coefficient (*R*)	*p* value	Standardized regression coefficient (β)	*p* value
Constant				<0.001 **
Years of education (year)	0.074	<0.001 **	0.117	<0.001 **
Annual household income (million yen/year)	0.101	<0.001 **	0.025	0.186
Age (years)			0.126	<0.001 **
Sex (male = 1, female = 2)			0.116	<0.001 **
Total energy intake (kJ/day)			0.614	<0.001 **

*p* Values and Pearson’s correlation Coefficients between intake levels and SES factiors, or *p* values and β (Standardized regression coefficient) showing the significance for linear regression analysis are present; * *p* < 0.05; ** *p* < 0.01.

When the subjects were classified into subgroups according to either education or income levels, age and sex ratio were significantly different by SES subgroups (Table 1, Table 2, *p* < 0.001). After adjusting for age and sex, the differences in SES factors, including years of education, proportion working as managers, and annual household income remained significantly (Table 1, Table 2). Among education subgroups, the K6 score was found to associated with education levels, the standardized regression coefficient (β) was −0.048, whereas the adjusted folate intake increased (β = 0.124). As to the income subgroups, there were significant positive associations in BMI and energy-adjusted folate intakes (β were 0.045 and 0.060), and negative associations in K6 score (β was −0.053). All of these results were adjusted for age and sex.

**Figure 1:**
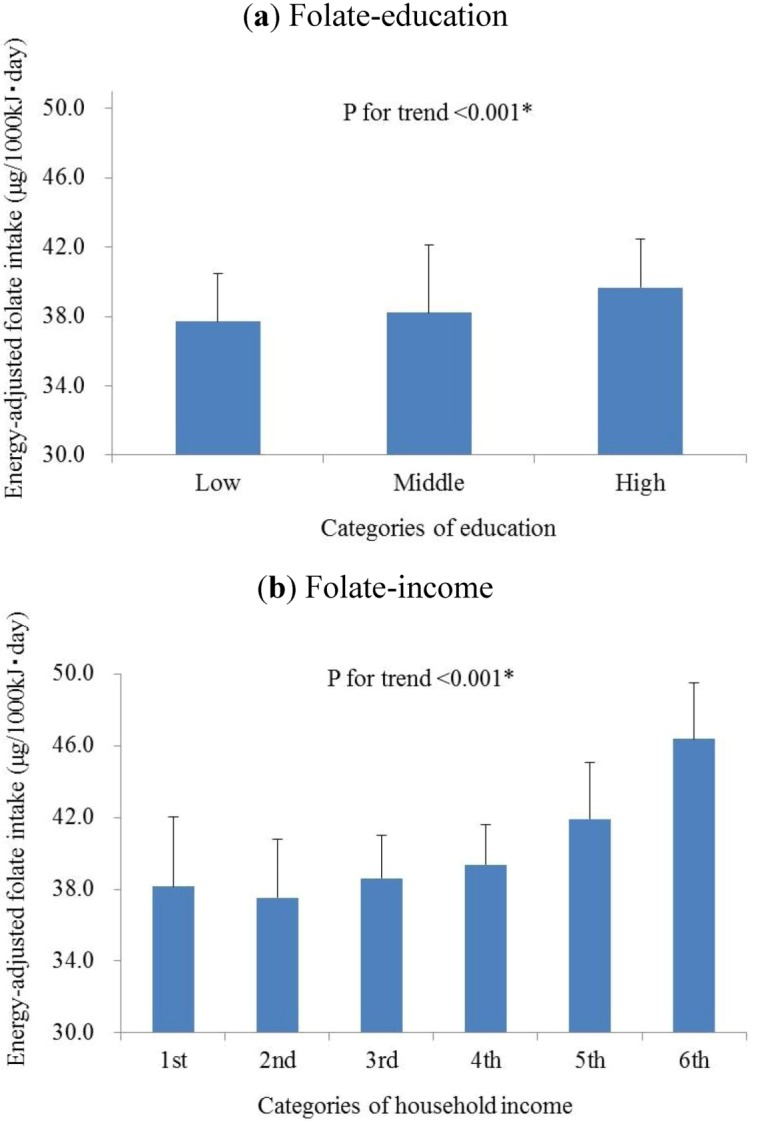
The associations of (**a**) education levels classified by the International Standard Classification of Education (ISCED) and (**b**) household incomes with folate intake levels. The classification of education subgroups is based on the International Standard Classification of Education (ISCED), approved by the United Nations Educational Scientific and Cultural Organization (UNESCO). Six subgroups are classified according to the self-reported household incomes of participants: 1, <3.0 million yen/year; 2, 3.0−4.99 million yen/year; 3, 5.0−7.99 million yen/year; 4, 8.0−9.99 million yen/year; 5, 10.0−15.0 million yen/year; 6, >15.0 million yen/year. Mean values of energy-adjusted folate intake and standard errors are present.

In a trend test in which SES factors were used as categorized variables, the age-, sex- and total energy intake-adjusted folate intake level of each subject was calculated, and the mean adjusted values were compared between subgroups. There were significant linear increases of the folate intake levels as the education (Figure 1a, *p* for trend <0.001) or income (Figure 1b, *p* for trend <0.001) increased.

We next assessed the effects of SES factors on intake levels by a multiple linear regression model. In this analysis, education level and annual household income were added at the same time, and age, sex and total energy intake were used as confounding factors. The results are shown in Table 3. Elder age, female gender, higher energy intake and higher education level (*p* < 0.001) were significantly associated with higher intake, but household income was not (*p* = 0.186). Considering the different dietary habits between genders, we also carried out a separate analysis for male and female subjects. In male subjects accounting for nearly 90% of the total, the results were similar to those of all subjects, education level was significantly associated (β = 0.123, *p* < 0.001), but household income was not (β = 0.022, *p* = 0.267). As to the women, neither education nor income was associated with folate intake (data not shown). The study may have been underpowered to detect a statistically significant association between folate intakes and education or income among women.

We also investigated whether the SES factors affect the health outcomes. As the results, the incidence of depressive symptoms (proportion of subjects with a K6 score ≥9) was negatively associated with education levels after controlling for age, sex, total energy intake and folate intake (ORs: 0.95, 95% CI: 0.91–1.00, *p* = 0.031), but not with household income. 

**Figure 2:**
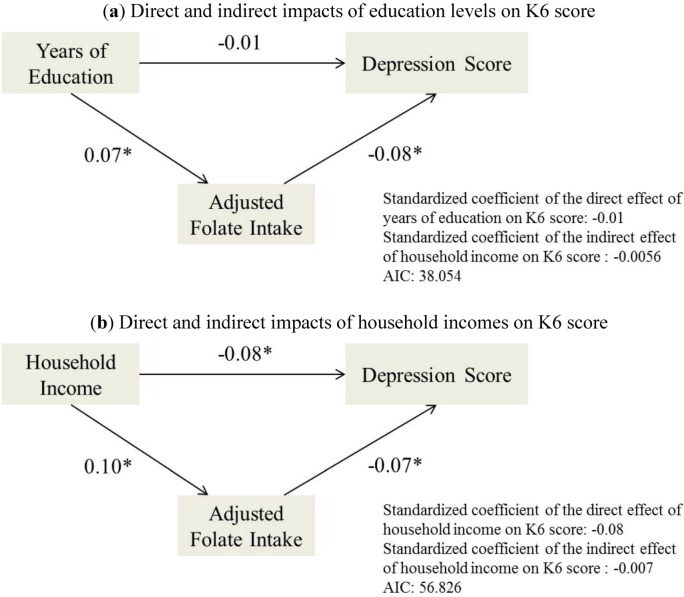
Path analysis of impact of folate intakes on depression scale (K6). Folate intakes are adjusted for total energy intakes. The direct standardized path coefficients are shown and the indirect coefficients are calculated representing the impact of the exogenous variables on the endogenous variables. The Akaike’s Information Criterions (AICs) are present and * indicates the significance level <0.05.

Results of path analysis are shown in Figure 2. In the relationship of education level with K6 score (Figure 2a), the direct standardized effect of years of education on depression scale (K6) was −0.01 (*p* = 0.737), the indirect standardized effect was −0.0056, which calculated by multiplied the coefficient of education on folate intake as 0.07 with that of folate intake on K6 as −0.08. The direct and indirect effect of household income K6 was −0.08 and −0.007 (Figure 2b), respectively.

## 4. Discussion

In the current study, we confirmed that there were statistically significant and linear associations between the dietary intake of folate and SES factors: years of education and household income (Figure 1) in Japanese workers. These findings are consistent with previous studies conducted in other countries, which reported separate associations of income and education onto folate intake [27,28,29,30,31,32]. Our data showed 36.4% subjects to have folate intakes below the Japanese RDA (240 μg/day). This level is lower than the total dietary intake of the USA population obtained from the National Health and Nutrition Examination Survey (NHANES) in 2003–2006 [39], only 36.8% (*n* = 833) meet the Estimated Average Requirement (EAR) as 320 μg/day, which was calculated for American adults [40], and 18.4% (*n* = 417) meet the 400 μg of RDA of USA [41]. Even compared with the relatively lower Japanese EAR as 200 μg/day, established by Japan’s Ministry of Health, Labour and Welfare in 2005 [42] there were 528 subjects (23.3%) who could not reach this standard, indicating the large room for nutritional intervention to relieve the SES effect on health outcomes.

Markedly, our results show the importance of education. After adjusting for age, sex, total energy intake and folate intake, education level was found to be negatively related to prevalence of depressive symptoms (defined as K6 ≥ 9, ORs (95% CI): 0.95 (0.91–1.00), *p* = 0.031, “Low” as the referent group). However, household income levels were not associated with these outcomes. In due consideration of the fact that raising income levels dramatically is quite difficult, education may have a realistic power to relieve the SES effect on health outcomes. Our results in general workers (both men and women) are consistent with them and provided new evidence for the importance of raising the education level in terms of understanding of human health.

We also found that education levels, but not household incomes, affect the prevalence of depressive symptom. These results are consistent with those reported by several study groups before 2001, in which a clear, graded association between educational attainment and reported depressive symptoms can be seen, demonstrating the consistency of this relationship [39,43,44,45]. On the other hand, a large-scale population-based survey conducted in Sweden [46], provided evidence that income was associated with depression, especially in women, whereas education was unrelated to depression in men and women overall. This disagreement was considered to be due to the differences of ethnicity, reflecting the different national character or economic and social development situation, as well as the fact that in our cohort the proportion of women is small.

However, whether SES factors affect the health outcomes directly or through other factors such as nutritional status, is unknown. In order to assess the role of the nutritional intake in the relationship between SES factors and health outcomes, we carried out a path analysis to detect the direct and indirect impacts of education and household income. In the education-folate intake-depression scale pathway, the direct and indirect impact of education on K6 score were −0.01 and −0.0056, respectively, over a half of the impact was accounted for by the indirect model, suggesting that the folate intake mediates the effects of education level on depression scale (Figure 2a). As to household income, the indirect impacts were less than 10% of the direct impacts, hence folate intake could not be thought as a mediator of the association between income and the depressive symptom.

Some limitations of the present study are worth mentioning. First, the cross-sectional nature of the study does not permit the assessment of causality owing to the uncertain temporality of the association. However, it is improbable that a low intake of folate would lead to low income or low education and the reverse causation is almost deniable. Second, our subjects were workers in one large company and not a random sample of Japanese workers, and thus the results may not apply to the general Japanese population. However, the workers were recruited from 22 offices all over Japan (From Hokkaido to Kyushu). So the geographical deviation was reasonably diluted, but it is noteworthy fact that our result is the result of a large company, not a small one. Thirdly, dietary data were obtained from a self-administered semi-quantitative dietary assessment questionnaire [36]. Because the actual dietary habits were not observed, the results should be interpreted with caution, although the validity of this questionnaire appears reasonable [38].

## 5. Conclusions

In the current study, the results of the structural equation modeling analysis have shown that in the pathway by which education level affects depression scale, the indirect effect mediated by the dietary folate intake was statistically significant, in addition, was considerable large that accounting for 56% of the direct effect. Education and income were strong and independent predictors of folate intake level in Japanese workers in general, and folate intake is an important factor mediating the association between education and depressive symptoms. Because it is difficult to raise one’s education and income level, nutritional interventions, which increase the folate intake, may alleviate the adverse impacts of the social disparity on mental health. 
